# Sleep duration, depressive symptoms, and postoperative delirium: a mediation analysis

**DOI:** 10.3389/fpsyt.2026.1796472

**Published:** 2026-04-27

**Authors:** Li-Heng Li, Hao Guo, Hao Wang, Yu-Bo Xie

**Affiliations:** 1 Department of Anesthesiology, Sichuan Clinical Research Center for Cancer, Sichuan Cancer Hospital & Institute, Sichuan Cancer Center, University of Electronic Science and Technology of China, Chengdu, China; 2 Department of Anesthesiology, Renmin Hospital, Hubei University of Medicine, Shiyan, China; 3 Department of Anesthesiology, The First Affiliated Hospital of Guangxi Medical University, Nanning, China

**Keywords:** depressive symptoms, geriatric assessment, mediation analysis, orthopedic surgery, perioperative care, postoperative delirium, sleep disturbance

## Abstract

**Purpose:**

The mechanism linking postoperative sleep duration to postoperative delirium (POD) remains unclear. This study examined the potential indirect pathway involving depressive symptoms (assessed via the PHQ-9) in the association between postoperative sleep duration and POD.

**Methods:**

We included 500 patients aged ≥ 65 years undergoing unilateral total hip or knee arthroplasty under general anesthesia. The associative pathways among sleep duration, depressive symptoms, and POD were assessed using covariate-adjusted three-step linear probability models with heteroscedasticity-consistent (HC3) robust standard errors, validated via 1000-resample bootstrapping and the Sobel test.

**Results:**

Postoperative sleep duration had significant total (β=-0.174, 95% CI [-0.190, -0.158]) and direct (β=-0.045, 95% CI [-0.057, -0.032]) associations with POD. An indirect pathway involving depressive symptoms was statistically significant (β=-0.129, 95% CI [-0.146, -0.113]). Adjusted paths for sleep duration→PHQ-9 (β=-2.660, 95% CI [-2.913, -2.407]) and PHQ-9→POD (β=0.049, 95% CI [0.045, 0.052]) were significant. Sensitivity analysis showed E-values of 1.67, 1.26, and 1.53 for total (OR = 0.840), direct (OR = 0.956), and indirect (OR = 0.879) pathways, respectively.

**Conclusion:**

Depressive symptoms are indirectly associated with the relationship between postoperative sleep duration and POD, alongside a direct independent association of sleep duration. Concurrent assessment and management of sleep and depressive symptoms are essential for optimizing perioperative care.

## Introduction

Globally, postoperative delirium (POD) represents a common and severe acute neurocognitive complication in elderly patients undergoing major surgery. POD is not only characterized by acute and fluctuating deficits in attention and awareness but is also associated with a substantial, nearly 3.5-fold increase in mortality and overall postoperative complication rates (1). Identifying modifiable risk factors and their underlying acute mechanisms during the perioperative period is therefore critical for developing effective prevention strategies (2, 3).

Depressive symptoms, common in older adults, have been increasingly recognized as a vulnerability factor for acute perioperative neurocognitive complications. Epidemiological studies indicate that preoperative and acute postoperative depressive symptoms are significantly associated with a higher risk of incident delirium in older surgical patients (4, 5). The biological mechanisms underlying this association may involve acute neuroinflammation, hypothalamic-pituitary-adrenal (HPA) axis dysregulation, and heightened surgical stress responses—all of which can precipitate acute neuronal dysfunction in brain regions critical for attention and awareness (6).

Sleep duration, a modifiable lifestyle factor, also acts as a key element in maintaining cognitive health. It is generally thought that normal daily sleep (roughly 7–8 hours) may support processes like memory consolidation, synaptic pruning, and the clearance of metabolic waste in the brain (7). However, abnormal sleep durations—including short sleep (fewer than 6 hours per day) and long sleep (more than 9 hours per day)—are quite prevalent among older adults. For instance, a considerable portion of the elderly population in the U.S. report short sleep, and another notable share have long sleep habits (8). Prior studies have suggested that short sleep might be linked to a notably higher risk of depressive symptoms and an increased likelihood of acute delirium; similarly, abnormal sleep duration also appears associated with elevated depressive symptoms and poorer performance in perioperative cognitive screening (9–11). Despite growing evidence linking sleep duration to depressive symptoms, depressive symptoms to POD, and sleep duration to POD individually, critical gaps remain in understanding the tripartite relationship between these factors in the acute surgical setting. First, most studies have focused on pairwise associations and under-explored the potential indirect pathways involving depressive symptoms. It remains unclear whether abnormal sleep duration is associated with POD risk independently or indirectly alongside elevated depressive symptoms, a question with important implications for perioperative intervention design (12–15).

## Materials and methods

### Study design and patient selection

This prospective cohort study enrolled elderly patients undergoing elective primary unilateral THA or TKA under general anesthesia at the First Affiliated Hospital of Guangxi Medical University between May, 2023, and October, 2024. The study protocol adhered to the Declaration of Helsinki and followed the STROBE guidelines. Ethical approval was obtained from the Ethics Committee Board of the First Affiliated Hospital of Guangxi Medical University, Nanning, China (No. 2023-K182-01), and the trial was registered prior to patient enrollment at Chinese Clinical Trial Registry (ChiCTR2300074920).

Prior to participation, written informed consent was obtained from all eligible subjects or their legal representatives. Eligible patients were aged ≥ 65 years and diagnosed with osteoarthritis or femoral head necrosis requiring primary THA or TKA. Exclusion criteria included: (1) refusal to participate, (2) recent history (within 6 months) of substance abuse, defined as illicit drug use or misuse of opioids/benzodiazepines, (3) severe cognitive impairment such as dementia or communication barriers, (4) admission to the intensive care unit (ICU) postoperatively, and (5) revision surgeries or non-primary complex joint arthroplasty procedures.

The target sample size was pre-determined prior to data collection based on anticipated effect sizes and previously published incidence rates of POD in comparable cohorts, with an initial recruitment of 780 patients; following rigorous post-collection quality screening and eligibility assessment, a total of 500 patients were ultimately included in the final analyses. A comprehensive quality check subsequently confirmed that the study dataset was complete, with no missing data for any primary or covariate variables included in the analyses.

### Clinical data collection

A standardized 60 minutes baseline evaluation was conducted by trained research assistants prior to surgery. The following preoperative variables were recorded:

Demographic and clinical variables: Preoperative assessments included age, sex, body mass index (BMI), smoking and alcohol use, living situation, and education level. Medical history was recorded for hypertension, diabetes mellitus, coronary artery disease, cerebrovascular disease, and metabolic syndrome (Mets), the latter defined by the presence of at least three of the following: obesity, elevated fasting triglycerides (TG), reduced high-density lipoprotein cholesterol (HDL-C), elevated blood pressure, and elevated fasting glucose (Glu) (16). Surgical type (THA or TKA) and ASA physical status were also documented. Frailty was evaluated using the FRAIL scale, assessing fatigue, resistance, ambulation, illnesses, and weight loss, and categorized as non-frail (score = 0), pre-frail (score 1–2), or frail (score > 2) (17). Cognitive function was screened using the Mini-Cog, with scores < 2 indicating mild impairment (18). Preoperative sleep complaints, anxiety, and depression were assessed using the Athens Insomnia Scale (AIS), Generalized Anxiety Disorder-7 (GAD-7), and Patient Health Questionnaire-9 (PHQ-9) (19), respectively. Pain intensity was measured using the Visual Analog Scale (VAS) at rest.

Laboratory parameters: Preoperative laboratory tests were obtained within 72 hours before surgery. The systemic immune-inflammation index (SII) was calculated using the most recent complete blood count values as: platelet count × neutrophil count/lymphocyte count. Additional laboratory variables included hemoglobin, hematocrit, white cell subtypes, glucose, C-reactive protein (CRP), albumin (ALB), TG, HDL-C, lipoprotein(a), and serum electrolytes (Na^+^, K^+^, Ca²^+^).

Intraoperative variables: Surgical and anesthetic details included operation type (THA or TKA), duration of surgery and anesthesia, estimated blood loss, total fluid infusion volume, intraoperative blood transfusion, and use of adjunct agents. General anesthesia was maintained with sevoflurane inhalation and a target-controlled infusion of either propofol or ciprofol combined with remifentanil. Intraoperative opioid dosages were standardized to fentanyl-equivalent doses. Hypotension was defined as a ≥ 20% reduction from baseline MAP or MAP < 60 mmHg.

Postoperative Analgesia and Rehabilitation: Postoperative analgesia included the use of patient-controlled analgesia (PCA), peripheral nerve blocks (PNB), and whether dexmedetomidine was incorporated into the PCA regimen. Multimodal analgesia protocols included scheduled NSAIDs, regional blocks, and rescue opioids. Sedative-hypnotics such as estazolam were only prescribed in cases of severe sleep disturbance and were not excluded from the final analysis to preserve generalizability.

Postoperative sleep duration was strictly defined as the patient’s self-reported total hours of sleep accumulated specifically during the night preceding the postoperative day 3 assessment. Furthermore, postoperative sleep disturbance (PSD) was operationalized as a distinct binary construct indicating subjective poor sleep quality or frequent awakenings, which is conceptually distinct from both the continuous measurement of postoperative sleep duration and the preoperative baseline chronic insomnia assessed via the Athens Insomnia Scale (AIS). To systematically assess the incidence of POD, the 3D-CAM (3-Minute Diagnostic Interview for Confusion Assessment Method) scale, an abbreviated and validated tool widely used for rapid delirium screening, was adopted. Delirium was diagnosed based on the scale’s adherence to the Diagnostic and Statistical Manual of Mental Disorders criteria, specifically requiring the presence of four key features: acute onset and fluctuating course of symptoms, inattention, disorganized thinking, and altered level of consciousness, with all criteria verified through the 3D-CAM’s structured interview items (20–25). While patients were monitored postoperatively, all scale assessment scores (including PHQ-9 and sleep parameters) and laboratory test results included in this specific analysis were cross-sectionally extracted based on data obtained on the third day after the surgery.

### Statistical analysis

#### Baseline data analysis

Continuous variables were tested for normality and presented as mean ± standard deviation or median (IQR), depending on distribution. Student’s t-test or Mann–Whitney U test was used for comparisons. Categorical variables were reported as counts (%) and analyzed using chi-square or Fisher’s exact tests. Statistical significance was defined as a two-tailed p-value of less than 0.05.

### Mediation analysis

The associative pathway involving depressive symptoms in the relationship between sleep duration and POD was evaluated using a three-step linear probability model (LPM) framework, incorporating heteroscedasticity-consistent (HC3) robust standard errors to appropriately handle the binary nature of the outcome variable. The LPM was specifically preferred over logistic approaches to provide directly interpretable absolute risk differences (proportions) and clinically meaningful effect sizes for the pathways. Model diagnostics, calibration, and fit indices (including variance explained) were evaluated to ensure model validity. To account for potential U-shaped or non-linear relationships between sleep duration and outcomes as suggested by previous literature, non-linear terms including restricted cubic splines and clinically meaningful sleep categorizations were initially tested. Furthermore, the covariate strategy was theoretically guided by a causal framework utilizing Directed Acyclic Graphs (DAGs) to identify true pre- and perioperative confounders of the exposure-mediator and mediator-outcome relationships (26–28), rather than relying solely on statistical collinearity thresholds, thereby avoiding the introduction of collider bias. First, the total effect model regresses cognitive outcome on sleep duration and selected covariates to quantify the overall association of sleep duration on cognition.

Second, the path model regresses depressive symptoms on sleep duration and covariates to quantify the effect of sleep duration on depressive symptoms. Third, the direct/indirect effect model regresses cognitive outcome on sleep duration, depressive symptoms and covariates to quantify the direct effect of sleep duration on cognition and the effect of depressive symptoms on cognition; the indirect effect is the product of a-path and b-path coefficients. The significance was confirmed via 1000-resample bootstrapping (95% confidence interval [CI] not overlapping 0) and the Sobel test (two-tailed p < 0.05). Last, Sensitivity analysis was performed by calculating the E-value on an odds ratio scale using the VanderWeele formula. This quantifies the minimum strength of association that an unmeasured confounder would need to have with both the predictor and the outcome to fully explain away the observed effect.

## Results

### Patient enrollment and baseline characteristics

**Figure 1:**
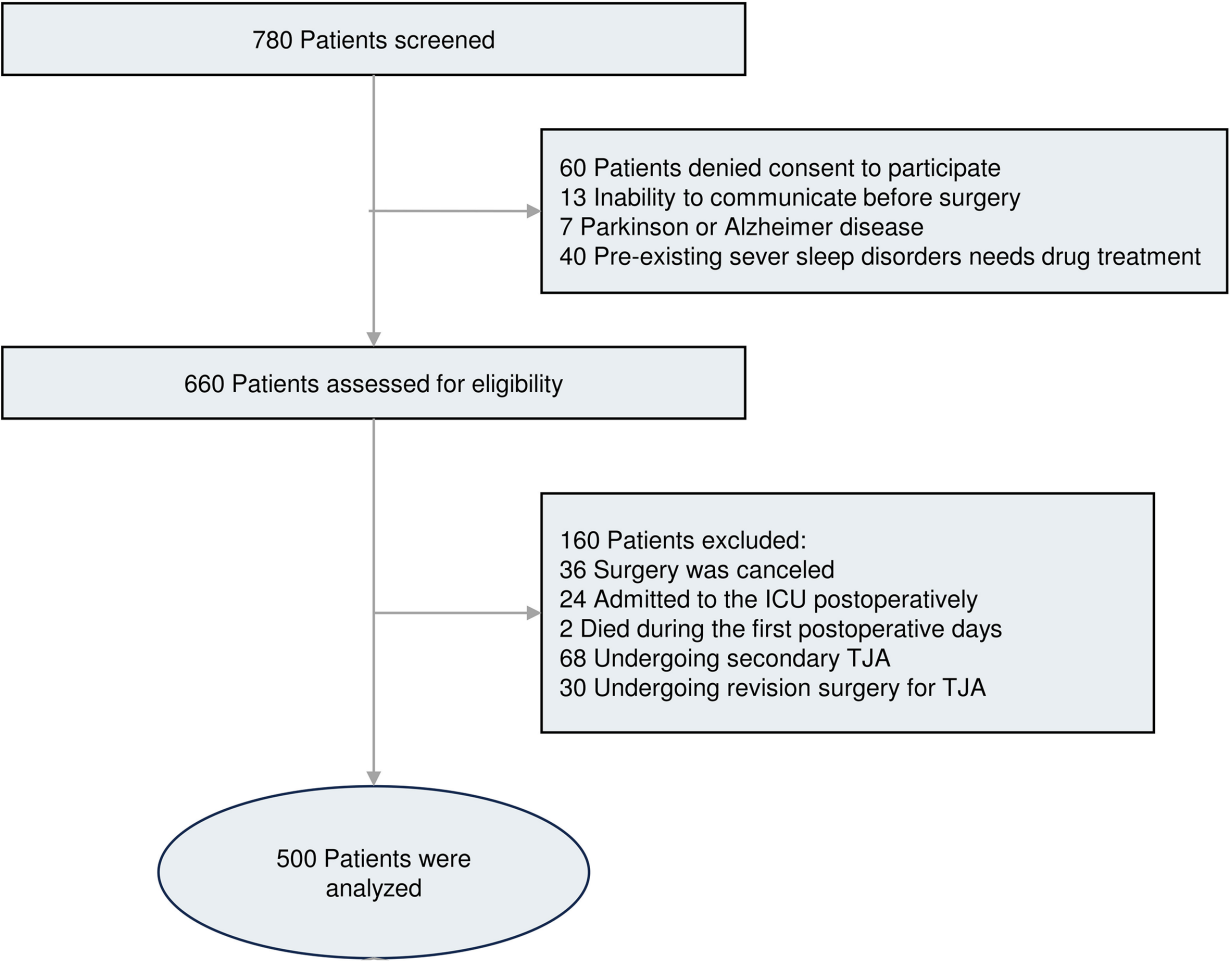
Diagram of study participant flow.

**Figure 2:**
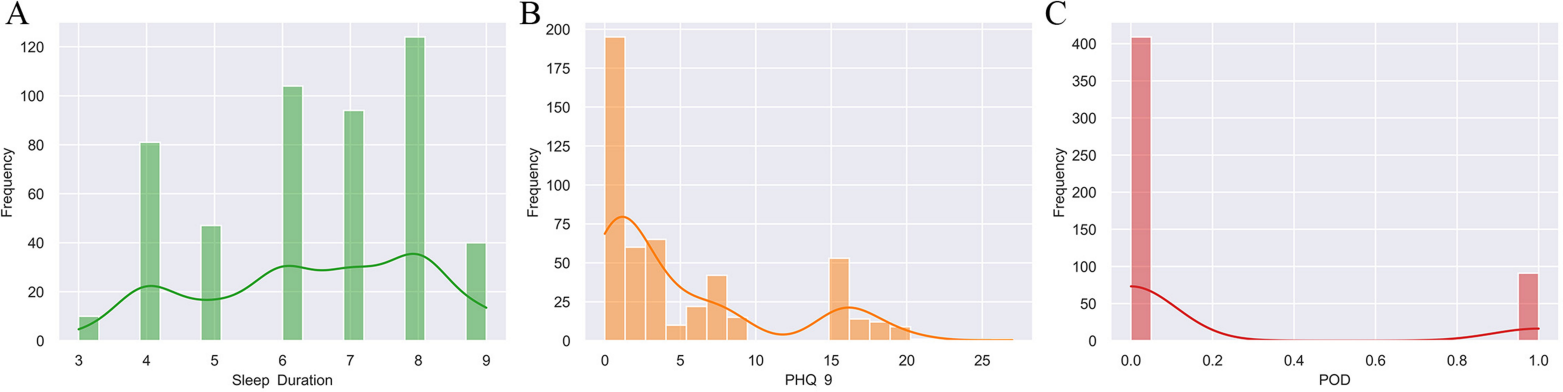
Distributions of core factor-related baseline data included in the analysis. Distributions of Sleep Duration (hours, **(A)**), Depressive Symptoms Score (PHQ-9, **(B)**), and POD **(C)** related measures among the baseline data included in the analysis.

Between May 2023 and October 2024, 780 elderly patients scheduled for elective THA or TKA under general anesthesia were screened. After applying inclusion and exclusion criteria, 500 patients were included in the final analysis (**Figure 1**). Of these, 91 (18.2%) developed POD during the first 72 hours after surgery. Baseline characteristics of the cohort are summarized in **Table 1**. **Figure 2** shows distributions of core factor related baseline data.

**Table 1:** Perioperative characteristics of patients.

Variates	non-POD (n=409)	POD (n=91)	*P* -value
Age	70.00[67.00-74.00]	72.00[68.50-76.00]	**0.004**
Gender Female	278 (68.0%)	66 (72.5%)	0.469
Male	131 (32.0%)	25 (27.5%)	
BMI	24.80 [22.70-27.30]	24.70 [22.20-27.10]	0.453
ASA II	249 (60.9%)	51 (56.0%)	0.463
III	160 (39.1%)	40 (44.0%)	
Education years ≤6	175 (42.8%)	43 (47.3%)	0.738
6-12	204 (49.9%)	42 (46.2%)	
≥12	30 (7.3%)	6 (6.6%)	
Drink			
No	356 (87.0%)	82 (90.1%)	0.530
Yes	53 (13.0%)	9 (9.9%)	
Smoke	370 (90.5%)	82 (90.1%)	0.999
Yes	39 (9.5%)	9 (9.9%)	
Frail status			0.845
Robust	87 (21.3%)	18 (19.8%)	
Pre-frail	234 (57.2%)	51 (56.0%)	
Frail	88 (21.5%)	22 (24.2%)	
VAS (Preop)	2.00 [2.00-3.00]	2.00 [2.00-3.00]	0.898
SII (Preop)	578.70 [409.20-826.10]	705.90 [405.00-929.80]	**0.042**
Anxiety (Preop)			
No	318 (77.8%)	74 (81.3%)	0.544
Yes	91 (22.2%)	17 (18.7%)	
Sleep disorder (Preop)			
No	262 (64.1%)	57 (62.6%)	0.893
Yes	147 (35.9%)	34 (37.4%)	
Cerebrovascular Disease			
No	377 (92.2%)	84 (92.3%)	0.999
Yes	32 (7.8%)	7 (7.7%)	
Coronary Heart Disease			
No	377 (92.2%)	87 (95.6%)	0.358
Yes	32 (7.8%)	4 (4.4%)	
Dexmedetomidine			
No	188 (46.0%)	46 (50.5%)	0.499
Yes	221 (54.0%)	45 (49.5%)	
Diabetes			
No	357 (87.3%)	80 (87.9%)	0.999
Yes	52 (12.7%)	11 (12.1%)	
Hypertension			
No	222 (54.3%)	47 (51.6%)	0.735
Yes	187 (45.7%)	44 (48.4%)	
Intraoperative hypotension			
No	81 (19.8%)	9 (9.9%)	**0.038**
Yes	328 (80.2%)	82 (90.1%)	
Metabolic Syndrome			
No	269 (65.8%)	59 (64.8%)	0.962
Yes	140 (34.2%)	32 (35.2%)	
Surgery duration	97.00 [81.00-115.00]	99.00 [81.50-120.00]	0.445
Bleeding volume	50.00 [50.00-200.00]	100.00 [50.00-200.00]	0.242
Infusion volume	1240.00 [1030.00-1600.00]	1360.00 [1110.50-1632.00]	0.081
Transfusion			
No	271 (66.3%)	55 (60.4%)	0.351
Yes	138 (33.7%)	36 (39.6%)	
PCA			
No	83 (20.3%)	26 (28.6%)	0.112
Yes	326 (79.7%)	65 (71.4%)	
PONV			
No	269 (65.8%)	42 (46.2%)	**<0.001**
Yes	140 (34.2%)	49 (53.8%)	
PSD			
No	198 (48.4%)	43 (47.3%)	0.933
Yes	211 (51.6%)	48 (52.7%)	
PHQ-9 (PO)	2.00 [1.00-4.00]	16.00 [15.00-18.00]	**<0.001**
Sleep Duration (PO)	7.00 [6.00-8.00]	4.00 [4.00-4.00]	**<0.001**

SII, systemic immune-inflammation index; PCA, Patient-Controlled Analgesia; PONV, Postoperative Nausea and Vomiting; PSD, Postoperative Sleep Disturbance; PO, Post Operation. Statistically significant P-values are marked in bold.

### Dynamic covariate selection for POD

**Figure 3:**
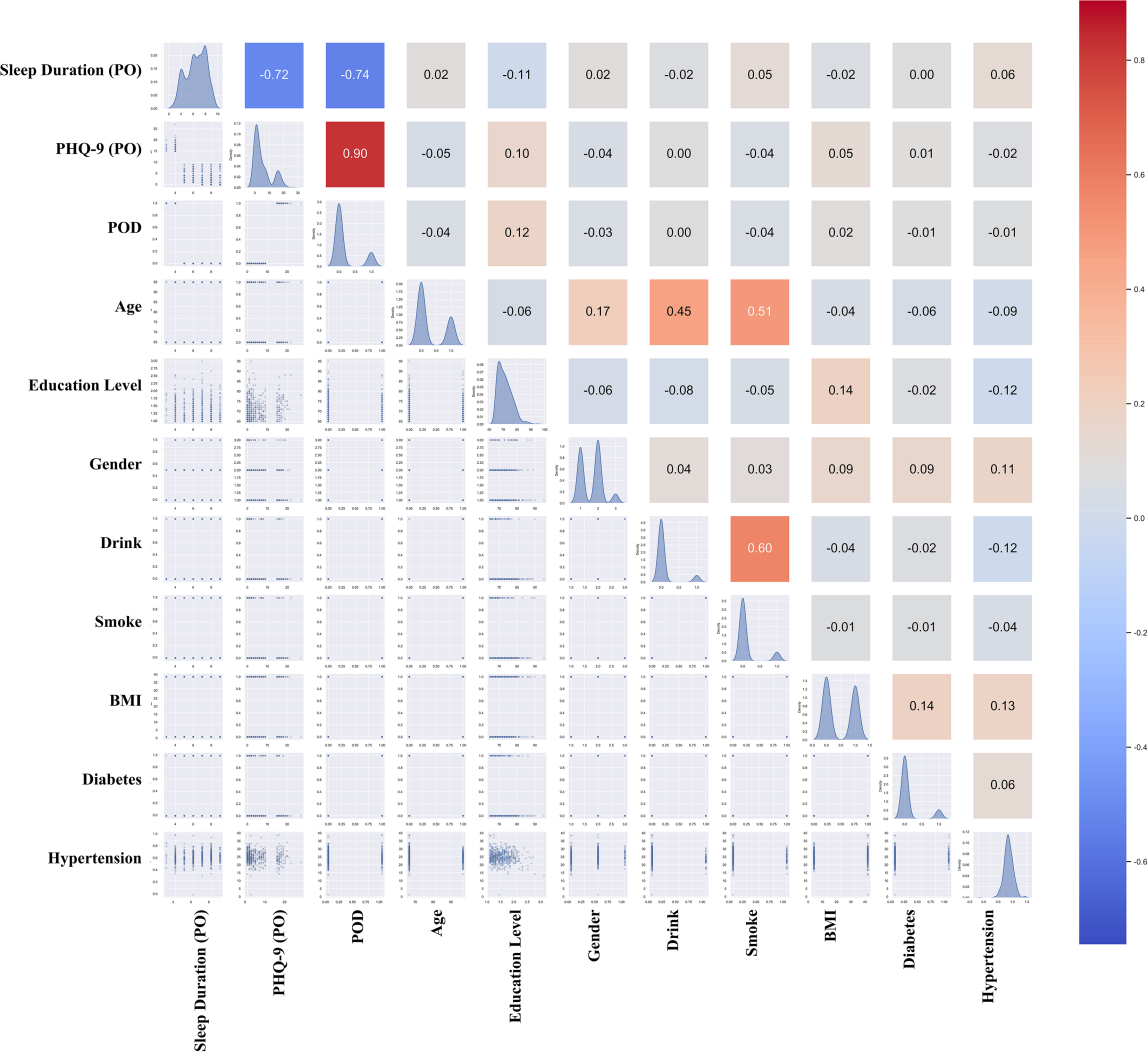
Correlation matrix of sleep duration, PHQ-9, POD, and covariates. This correlation matrix displays the pairwise relationships among sleep duration, total depressive symptoms score (PHQ-9), POD, and selected covariates. The upper triangle presents Pearson correlation coefficients, with colors indicating the strength and direction of correlations (red for positive, blue for negative, and intensity reflecting magnitude). The lower triangle shows scatter plots or density distributions for each variable pair, illustrating bivariate relationships visually.

Following the causal framework to identify theoretically relevant confounders (such as age, preoperative cognitive status, and surgical duration), dynamic covariate selection was performed to refine the model transparency. Potential multicollinearity among theoretically relevant variables, including preoperative sleep disorders, preoperative anxiety, and frailty, was rigorously assessed. Diagnostic tests confirmed the absence of severe multicollinearity among these constructs, with Variance Inflation Factor (VIF) values of 1.182 for preoperative sleep disorders, 1.199 for preoperative anxiety, and 1.067 for frailty (all well below the threshold of 10). To avoid collider bias and over-adjustment, the final full model retained the most robust true confounders (Age, PONV, Ca²⁺, and PCA) to maintain statistical stability without sacrificing true confounders. **Figure 3** presents Pearson correlation coefficients in its upper triangle, where colors encode the direction and strength of correlations.

The lower triangle displays bivariate scatter plots alongside density distributions. All illustrating the relationships among sleep duration, PHQ-9, POD, and the covariates retained after selection, those excluded for high collinearity have been omitted.

### Mediation analysis

This study investigated the indirect pathway involving the PHQ-9 depression score in the relationship between sleep duration and POD through a three-step linear probability model. The final model demonstrated excellent fit with an R-squared value of 0.8405, indicating that the included predictors accounted for approximately 84.05% of the variance in the risk of POD. Furthermore, preliminary analyses incorporating restricted cubic splines and clinical categorizations did not indicate a statistically significant non-linear or U-shaped relationship between postoperative sleep duration and POD risk. Therefore, the final models utilized sleep duration as a continuous linear predictor to preserve model parsimony and facilitate the clinical interpretation of the absolute risk differences. Model diagnostics, incorporating heteroscedasticity-consistent (HC3) robust standard errors, confirmed appropriate calibration for absolute risk estimation. The detailed results showed that:

Mediation analysis demonstrated that postoperative sleep duration was negatively associated with POD, exerting a significant total effect (β= -0.174, 95% CI: [-0.190, -0.158]) and direct effect (β= -0.045, 95% CI: [-0.057, -0.032]). Additionally, the PHQ-9 depression score formed a significant indirect pathway in this relationship (β= -0.129, 95% CI: [-0.146, -0.113]). This indirect pathway accounted for approximately 74.1% (-0.129/-0.174) of the total effect. In terms of absolute risk interpretation, the effect sizes demonstrate a clinically meaningful reduction in the probability of POD associated with better sleep duration. Specifically, shorter sleep duration significantly predicted higher depressive symptoms (Path a: β= -2.660, 95% CI: [-2.913, -2.407]), and higher depressive symptoms were in turn positively associated with the occurrence of POD (Path b: β= 0.049, 95% CI: [0.045, 0.052]).

**Figure 4:**
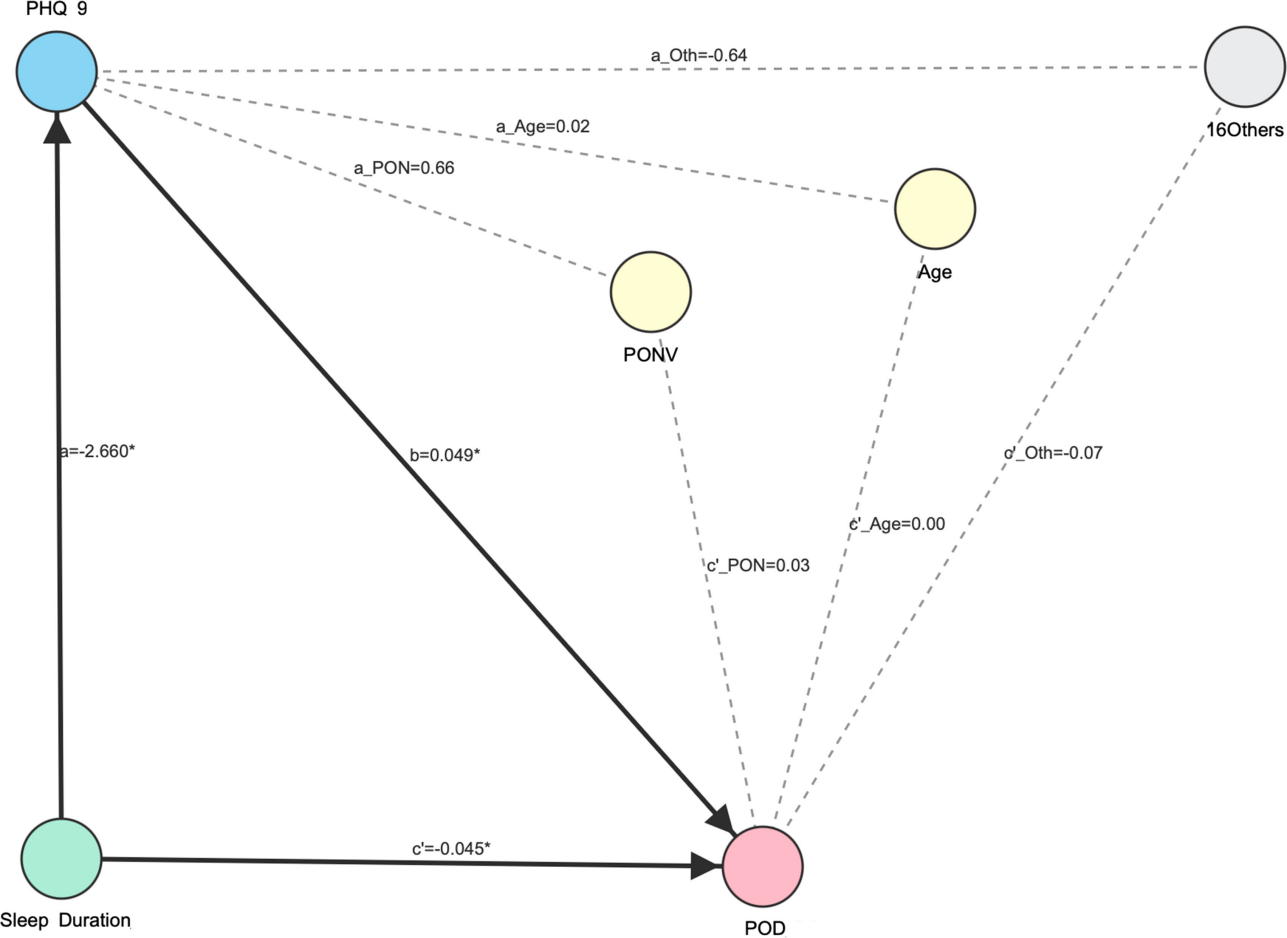
Mediation model of sleep duration on POD via PHQ-9 score. This mediation analysis model presents the influence paths and effect values of Sleep Duration (PO) and PHQ-9 (PO) on POD, with PONV, Age, and Others included as covariates; solid lines represent statistically significant paths, and dashed lines represent statistically insignificant paths.

All paths remained statistically significant after adjusting for key clinical covariates (**Table 2**; **Figure 4**). Of the covariates, only the two most highly correlated ones are presented; the remaining covariates, categorized as “others,” showed no interference or effect in this mediation analysis model.

**Table 2:** Mediation path statistics for sleep duration, PHQ-9, and POD.

Pathway type		Estimated value	95% CI	P-value
Total association (c)	Sleep Duration →POD	-0.174	[-0.190, -0.158]	<0.001
Path a	Sleep Duration →PHQ 9	-2.660	[-2.913, -2.407]	<0.001
Path b	PHQ 9 →POD	0.049	[0.045, 0.052]	<0.001
Direct pathway (c’)	Sleep Duration →POD	-0.045	[-0.057, -0.032]	<0.001
Indirect pathway (a×b)	Sleep Duration →PHQ 9 →POD	-0.129	[-0.146, -0.113]	<0.001

For the indirect effects, the 95% confidence intervals derived from Bootstrap do not contain 0, suggesting significant mediation effects. To highlight the primary findings, this table specifically reports the core mediation paths. The model was fully adjusted for covariates (PONV, Age, and Others) as visually detailed with their respective path coefficients in **Figure 4**. Additionally, minor mathematical discrepancies between the exact product of the displayed Path a and Path b coefficients and the final reported indirect effect are strictly due to rounding to three decimal places for presentation purposes; all final effect sizes were calculated using exact, unrounded statistical computations.

### Sensitivity analysis of mediation effect

A sensitivity analysis was conducted to evaluate the robustness of the mediation effects (**Table 3**). For the total effect of sleep duration on postoperative delirium, the OR was 0.840, with an E-value of 1.67. For the direct effect of sleep duration on postoperative delirium with depressive symptoms controlled, the OR was 0.956, with an E-value of 1.26. For the indirect effect of sleep duration on postoperative delirium via depressive symptoms, the OR was 0.879, with an E-value of 1.53. Unobserved confounders would need to enhance the associations by these respective minimum strengths to offset the observed effects.

**Table 3:** Sensitivity analysis results of mediation pathways.

Effect type		Effect OR value	E - value
Total effect (c)	Sleep Duration→POD	0.84	1.67
Direct effect (c’)	Sleep Duration→POD	0.956	1.26
Indirect effect (a×b)	Sleep Duration→PHQ 9→POD	0.879	1.53

The E-value (Minimum Confounding Strength) quantifies the minimum strength of association, on the odds ratio scale, that an unmeasured confounder would need to have with both the predictor and the outcome to fully explain away the observed effect. Unobserved confounders would need to enhance the associations by these respective minimum strengths to offset the observed total, direct, and indirect effects.

## Discussion

This study identified a robust association between sleep duration and POD, highlighting a significant indirect pathway involving the PHQ-9 depression score alongside a direct relationship. Important pre- and perioperative confounders were adjusted for without invalidating this associative pathway. The core contribution of this study lies in clarifying the dual pathways through which sleep duration is linked to POD risk in older adults.

First, sleep duration exerted a significant direct effect on POD. This aligns with the biological mechanisms proposed in prior research (7, 10, 29): normal sleep supports cognitive maintenance by facilitating memory consolidation, synaptic pruning, and metabolic waste clearance via the glymphatic system. Abnormal postoperative sleep duration may acutely disrupt these processes, leading to direct impairments in brain regions critical for attention and awareness. The direct effect on acute cognitive fluctuations suggests that cortical networks may be highly sensitive to sleep disturbances, which is consistent with evidence that processing speed relies on efficient neural signaling, a function highly vulnerable to sleep-related disruptions in neurotransmitter balance (30–34).

Second, the study identified depressive symptoms as a key component of the indirect pathway linking sleep duration to POD. This indirect association indicates that abnormal sleep duration may exacerbate depressive symptoms, which then contribute to the onset of acute delirium.

Notably, the positive value of the indirect effect suggests that depression may partially offset or modify the direct impact of sleep on cognition, highlighting the complexity of their interplay. For instance, short sleep may directly impair cognitive reserve but also increase the severity of depressive symptoms, which independently precipitates POD, resulting in a more severe overall acute effect than either factor alone (6, 35).

Our findings build on and advance prior research in critical ways. This addresses the longstanding gap in understanding how these three factors interact, moving beyond fragmented pairwise analyses to a more integrated model. Second, the study overcomes key methodological limitations of previous work. The retention of potential covariates in dynamic variable selection confirms that these confounders are non-negligible, and their inclusion enhances the robustness of our mediation results. This stands in contrast to smaller, regional studies that often fail to adjust for such factors, leading to potential overestimation of effect sizes. Third, the study confirms the clinical relevance of modifiable factors (sleep, depressive symptoms) for perioperative neurocognitive outcomes. Prior research (11, 36–38) has established that short sleep increases depressive symptom severity and delirium risk, but our findings clarify that targeting depressive symptoms may be a viable intervention point to mitigate the acute cognitive impact of poor sleep. For example, improving sleep hygiene to normalize sleep duration could reduce depressive symptoms, which in turn may reduce the risk of POD, offering a two-pronged strategy for prevention (39, 40).

The results have important implications for addressing acute perioperative neurocognitive complications. Clinically, the findings support the integration of sleep and depressive symptom screening into routine perioperative geriatric care. For older adults scheduled for major surgery, assessing sleep duration and depressive symptoms could help identify modifiable contributors to POD. Interventions targeting both postoperative sleep quality and depressive symptoms may be more effective than addressing either factor alone in the acute surgical setting. From a hospital-management perspective, the findings highlight the value of perioperative strategies to promote healthy sleep and mental well-being in older surgical patients. For instance, optimizing ward environments to normalize postoperative sleep duration, alongside targeted screening for depressive symptoms, could identify at-risk individuals early; such preventive efforts may also yield substantial economic benefits by reducing hospital length of stay and avoiding the need for extended post-acute care (12, 41, 42).

This study has several limitations. First, the observational design and the cross-sectional nature of measuring sleep duration, depressive symptoms, and POD on postoperative day 3 preclude definitive causal inference. The temporal sequence cannot be definitively established, and we cannot rule out reverse causality, where the onset of acute delirium or its prodromal phase directly disrupted sleep patterns prior to cognitive decline. Second, while the 3D-CAM is a validated tool, the gold standard for defining POD remains the CAM combined with a psychiatrist’s confirmation. Furthermore, relying heavily on an assessment on postoperative day 3 may miss early or subsequent delirium episodes due to the highly fluctuating nature of the syndrome. Third, assessing depressive symptoms via the PHQ-9 in patients with active delirium is challenging due to symptom overlap. Neurocognitive features of delirium, such as inattention, may be misconstrued as depressive symptoms, potentially artificially inflating scores in the POD group. Fourth, sleep duration was measured via self-report representing the night prior to assessment, which is susceptible to recall bias in the acute postoperative setting due to pain or medications. The lack of objective measures, such as actigraphy, limits precision. Fifth, while the indirect pathway accounted for a substantial proportion of the total effect (nearly 74%), the modest E-values (1.26 to 1.67) suggest that relatively weak unmeasured confounding could potentially explain the findings away, warranting a cautious interpretation of the model’s robustness. Finally, generalizability is limited because this was a single-center cohort of older adults undergoing elective unilateral THA/TKA under general anesthesia. Future multi-center longitudinal studies utilizing objective sleep monitoring, repeated continuous delirium assessments, and comprehensive psychiatric evaluations are needed to disentangle these intertwined acute symptoms.

## Conclusion

Our findings suggest that depressive symptoms form a significant indirect pathway in the association between postoperative sleep duration and POD in older adults, while also identifying a direct independent association between sleep duration and acute cognitive impairment. The results underscore the importance of assessing both sleep and depressive symptoms in efforts to prevent POD, offering actionable insights for clinical perioperative care.

## Data Availability

The original contributions presented in the study are included in the article/supplementary material. Further inquiries can be directed to the corresponding author.
